# Clinical analysis of peri-operative hidden blood loss of elderly patients with intertrochanteric fractures treated by unreamed proximal femoral nail anti-rotation

**DOI:** 10.1038/s41598-018-21703-4

**Published:** 2018-02-19

**Authors:** Bohua Li, Jun Li, Shanxi Wang, Lei Liu

**Affiliations:** Department of Orthopedics, West China Hospital, Sichuan University, 37# Wainan Guoxue Road, Chengdu, 610041 People’s Republic of China

## Abstract

The purpose of this study was to quantify the peri-operative blood loss of elderly patients with intertrochanteric fractures treated by unreamed proximal femoral nail anti-rotation (PFNA) and analyze whether the substantial hidden blood loss was induced by initial trauma or the operation. The clinical data of 123 patients with intertrochanteric fracture treated with unreamed PFNA from Jan 2013 to Apr 2017 were analyzed retrospectively. Blood routine on admission day (ADM), pre-operative day one (PRE), post-operative days one and three (POD1 and POD3) and the visible blood loss (VBL) were obtained. The total blood loss (TBL) from ADM to POD1 and POD3 were 693.5 ± 359.6 ml and 863.8 ± 429.9 ml, of which the corresponding hidden blood loss (HBL) was 86.8% and 89.4% respectively. The mean TBL and HBL from ADM to PRE (375.5 ± 242.0 ml, 375.5 ± 242.0 ml) were higher than that from PRE to POD1 (318.0 ± 183.4 ml, 226.5 ± 163.2 ml), p < 0.001 respectively. There was no significant difference between HBL from ADM to PRE and HBL from PRE to POD3 (375.5 ± 242.0 ml, 396.7 ± 254.0 ml, p = 0.361). The majority of peri-operative HBL occurred before surgery, it was mainly associated with the initial trauma rather than the operation.

## Introduction

With the rapid increase of the elderly population, intertrochanteric fractures have become a severe health issue^1,2^. Although conservative treatment can avoid surgical trauma, patients have to stay in bed for a long time, and there is a high rate of complications such as prolonged immobilization, hypostatic pneumonia, decubitus ulcers, lower extremity deep vein thrombosis, urinary system infection, coxa adducta, and limb shortening, which may lead to high morbidity and mortality^3–6^. Therefore, the surgical treatment is more preferable in most cases. Peri-operative blood lose is associated with a high mortality risk among anaemic elderly patients^2,7–10^. However, compared with the visible blood loss, the hidden blood loss is often ignored. Several studies have proved the relation between HBL and the process of standard reaming PFNA^10–13^.

We speculated that the majority of HBL occurred prior to the unreamed PFNA surgery. Thus, the aim of this study was to quantify the peri-operative blood loss especially the pre-operative part of elderly patients with intertrochanteric fractures treated by unreamed PFNA and analyze whether the substantial hidden blood loss was induced by initial trauma rather than the operation.

## Materials and Methods

This retrospective study was conducted at the Department of Orthopedic Surgery at West China Hospital, Sichuan University. Before the study began, we had carefully consulted the Ethics Committee and Institutional Review Board of West China Hospital, Sichuan University. They suggested that the unreamed PFNA was a very mature treatment in our hospital, this study was a retrospective analysis of previous clinical data, it did not involve special interventions for patients, and we should carry out this study in compliance with the Helsinki Declaration and provide informed consent to patients to let them know the way their data may be used. So, this study was approved by Ethics Committee and Institutional Review Board of West China Hospital, Sichuan University. Accordingly, written informed consent from all the participants was obtained. The clinical study was performed in accordance with the Declaration of Helsinki on ethical principles for medical research involving human subjects.

### Inclusion and exclusion criteria

The inclusion criteria were patients over 65 years old with an intertrochanteric fracture, ability to walk independently without aids before fracture. The exclusion criteria included the inability to walk before injury, an American Society of Anaesthesiologists (ASA) score of V, pathologic fractures or the presence of metastatic disease, polytrauma, open fractures, hematopathy, laboratory results of bleeding disorders, taking drugs like aspirin, vitamin K antagonists, platelet inhibitors that possibly impact blood coagulation, non-steroidal anti-inflammatory drugs, alcohol abuse, gastrointestinal hemorrhage, admission to hospital 12 h or more after trauma, time to surgery was or more than three weeks after the trauma, chronic or acute liver diseases, organ failure diseases, deaths.

### Intra- and post-operative treatment

All surgeries were performed under general anesthesia by one surgical team, the senior surgeon was experienced in dealing with intertrochanteric fractures. The technique followed standard protocols of PFNA in principle, but when the femur was exposed with the awl, the PFNA was carefully inserted without the reaming process. Drainage was placed in the incision according to the intra-operative bleeding condition and was removed 24 hours after operation in the light of the drainage condition. The total drainage of post-operation was recorded as visible blood loss (VBL). A single dose of low molecular weight heparin (LMWH; 2000 IU in 0.2 ml; Clexane, Sanofi-Aventis, France) was injected percutaneously every 24 hours after operation. The criteria for blood transfusion were an Hb level < 70 g/L or a level < 80 g/L when symptoms of anaemia present.

### Data collection

The hospital records contained data on sex, age, height, weight, body mass index(BMI), admission albumin (ALB) levels, Evans-Jensen classification, injury side, injury mechanism, time from injury to operation, duration of operation, the American Society of Anesthesiologists (ASA) scores, VBL. Blood routine of different time points including haematocrit (Hct) and haemoglobin (Hb) were obtained on admission day (ADM), pre-operatively day one(PRE), post-operative days one and three (POD1 and POD3).

### Blood loss calculation

VBL contained intraoperative blood loss and the volume of drainage after operation. Intraoperative visible blood loss corresponded to the amount of liquid in the suction bottle minus the amount of liquid used to flush the wound, and the total volume of blood lost in gauzes and surgical towels. The volume of drainage was obtained and measured as the post-operative visible blood loss. The transfused blood volume (BV_trans_) was also recorded.

TBL was calculated from the change in the Hct level and estimated patient’s blood volume (PBV). According to Nadler’s formula^14^, PBV (L) = k1 *height (m) ^3^ + k2 *weight (kg) + k3. For men, k1 = 0.3669, k2 = 0.03219, and k3 = 0.6041, and for women, k1 = 0.3561, k2 = 0.03308, and k3 = 0.1833. Generally, the total red blood cell loss (TRBCL) was calculated according to the Gross formula^15^: TRBCL (L) = PBV* (Hct_pre-operation_ − Hct_post-operation_), TBL (L) = TRBCL/ Hct_pre-operation_ + BV_trans_, HBL (L) = TBL − VBL.

According to the formula above, we calculated indexes of consecutive times as follows: TRBCL, TBL, HBL, BV_trans_ from admission to pre-operative day one (pre), pre-operative day one to post-operative day one (pod1) and post-operative day one to three (pod3).

$$\begin{array}{rcl}{{\rm{TBL}}}_{{\rm{pre}}}({\rm{L}}) & = & {{\rm{TRBCL}}}_{{\rm{pre}}}/{{\rm{Hct}}}_{{\rm{ADM}}}+{{\rm{BV}}}_{{\rm{trans}}{\rm{pre}}}\\  & = & {\mathrm{PBV}}^{\ast }\,({{\rm{Hct}}}_{{\rm{ADM}}}-{{\rm{Hct}}}_{{\rm{PRE}}})/{{\rm{Hct}}}_{{\rm{ADM}}}+{{\rm{BV}}}_{{\rm{trans}}{\rm{pre}}}\\  & = & {{\rm{HBL}}}_{{\rm{pre}}};\end{array}$$

$$\begin{array}{rcl}{{\rm{TBL}}}_{{\rm{pod1}}}({\rm{L}}) & = & {{\rm{TRBCL}}}_{{\rm{pod1}}}/{{\rm{Hct}}}_{{\rm{PRE}}}+{{\rm{BV}}}_{{\rm{trans}}{\rm{pod1}}}\\  & = & {{\mathrm{PBV}}^{\ast }(\mathrm{Hct}}_{{\rm{PRE}}}-{{\rm{Hct}}}_{{\rm{POD1}}})/{{\rm{Hct}}}_{{\rm{PRE}}}+{{\rm{BV}}}_{{\rm{trans}}{\rm{pod1}}};\end{array}$$

$$\begin{array}{rcl}{{\rm{TBL}}}_{{\rm{pod3}}}({\rm{L}}) & = & {{\rm{TRBCL}}}_{{\rm{pod3}}}/{{\rm{Hct}}}_{{\rm{POD1}}}+{{\rm{BV}}}_{{\rm{trans}}{\rm{pod3}}};\\  & = & {\mathrm{PBV}}^{\ast }\,({{\rm{Hct}}}_{{\rm{POD1}}}-{{\rm{Hct}}}_{{\rm{POD3}}})/{{\rm{Hct}}}_{{\rm{POD1}}}+{{\rm{BV}}}_{{\rm{trans}}{\rm{pod3}}};\\  & = & {{\rm{HBL}}}_{{\rm{pod3}}};\end{array}$$

$${{\rm{HBL}}}_{{\rm{pod1}}}({\rm{L}})={{\rm{TBL}}}_{{\rm{pod1}}}-{\rm{VBL}}{\rm{.}}$$

TBL and HBL from PRE to POD3 were calculated as TBL_post_ and HBL_post_.

$${{\rm{TBL}}}_{{\rm{post}}}({\rm{L}})={{\rm{TBL}}}_{{\rm{pod1}}}+{{\rm{TBL}}}_{{\rm{pod3}}};$$

$${{\rm{HBL}}}_{{\rm{post}}}({\rm{L}})={{\rm{HBL}}}_{{\rm{pod1}}}+{{\rm{HBL}}}_{{\rm{pod3}}}{\rm{.}}$$

TBL and HBL from ADM to POD1 and POD3 were calculated as TBL_all1_, TBL_all3_, HBL_all1_, and HBL_all3_.

$${{\rm{TBL}}}_{{\rm{all1}}}({\rm{L}})={{\rm{TBL}}}_{{\rm{pre}}}+{{\rm{TBL}}}_{{\rm{pod1}}};\,{{\rm{TBL}}}_{{\rm{all3}}}({\rm{L}})={{\rm{TBL}}}_{{\rm{all1}}}+{{\rm{TBL}}}_{{\rm{pod3}}};$$

$${{\rm{HBL}}}_{{\rm{all1}}}({\rm{L}})={{\rm{HBL}}}_{{\rm{pre}}}+{{\rm{HBL}}}_{{\rm{pod1}}};\,{{\rm{HBL}}}_{{\rm{all3}}}({\rm{L}})={{\rm{HBL}}}_{{\rm{all1}}}+{{\rm{HBL}}}_{{\rm{pod3}}}{\rm{.}}$$

### Statistical analysis

Statistical analyses were performed using SPSS Statistics version 24.0 software (SPSS Inc, Chicago, Illinois). Data were shown as the mean with 95% confidence intervals, and differences between different peri-operative time were analyzed using a one-way analysis of variance(ANOVA) with replicate measures. The level of significance was set at p < 0.01 for all statistical analysis.

## Results

**Figure 1:**
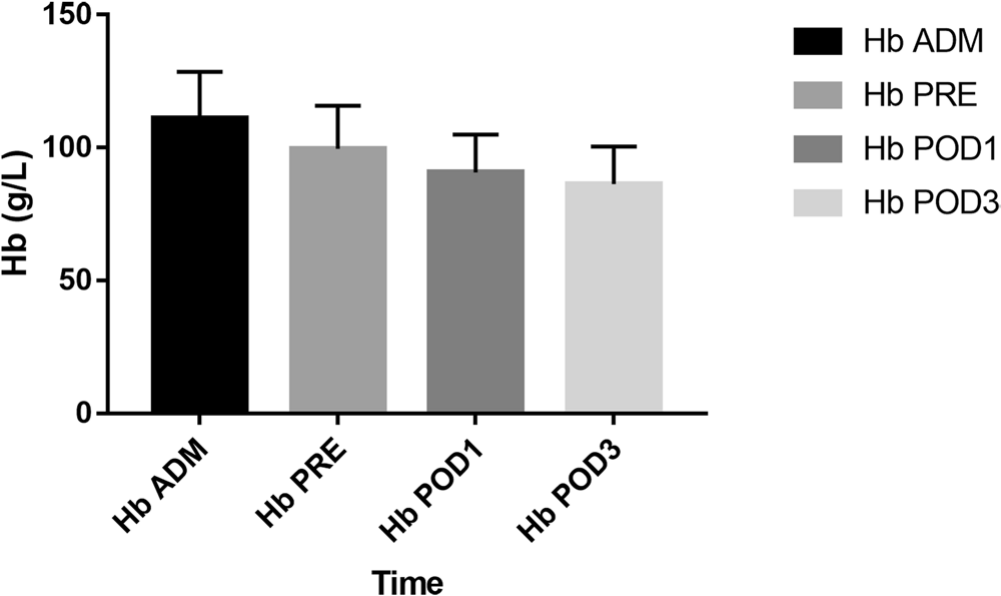
The Hb droped from admission to post-operative day three.

A total 123 patients who met the criteria were included in this study, their details were presented in Table 1. Apparently, we could find that there was a large drop in Hb of these elderly patients even before surgery (Fig. 1). We calculated details of their peri-operative blood loss to get conclusions which were described in Table 2.

**Table 1:** Details of the 123 patients with intertrochanteric fractures assessed for peri-operative blood loss.

**Total patients number**	123
Gender	
Male	53
Female	70
Age(years)	77.67 ± 9.36 (45 to 94)
BMI (kg/m²)	22.19 ± 3.21 (15.21 to 31.22)
Admission ALB (g/L)	37.8 ± 4.14 (27.70 to 48.30)
Evans-Jensen classification	
Type-I	3
Type-II	14
Type-III	31
Type-IV	46
Type-V	29
Side of injured	
Left	75
Right	48
Injury mechanism	
Fall from a stand height	113
Traffic accident	100
Time to operation (days)	6.44 ± 2.88 (2 to 14)
Operative time (min)	49.98 ± 8.60 (30 to 65)
ASA classification	
ASA I	3
ASA II	44
ASA III	76

**Table 2:** Peri-operative blood loss details.

**HBL(ml)**	**pre** 375.5 ± 242.0	**pod1**	226.5 ± 163.2	**post** 396.7 ± 254.0
**TBL(ml)**	**pre** 375.5 ± 242.0	**pod1**	318.0 ± 183.4	**post** 488.2 ± 271.1
**VBL(ml)**	91.5 ± 54.6			
**TBL**_**all1**_ **(ml)**	693.5 ± 359.6	**TBL**_**all3**_ **(ml)**	863.8 ± 429.9	
**HBL**_**all1**_ **(ml)**	602.0 ± 356.2	**HBL**_**all3**_ **(ml)**	772.3 ± 424.7	
**Transfusion rate (%)** 14.6(18/123)				

The HBL_all1_ was 602.0 ± 356.2 mL, which was 86.8% of TBL_all1_ (693.5 ± 359.6 ml). The HBL_all3_ was 772.3 ± 424.7 ml, which was 89.4% of TBL_all3_ (863.8 ± 429.9 ml). The HBL_pre_ was 375.5 ± 242.0 ml, which was 62.4% of HBL_all1_ (602.0 ± 356.2 ml) and 48.6% of HBL_all3_ (772.3 ± 424.7 ml). The VBL was only 91.5 ± 54.6 ml. Three and six patients respectively required pre-and post-operative blood transfusions due to low Hb levels. Nine patients received intra-operative blood transfusions due to low blood pressure. The blood transfusion rate was 14.6% (18/123).

The mean TBL_pre_ (375.5 ± 242.0 ml) were higher than TBL_pod1_ (318.0 ± 183.4 ml), but it was not statistically significant (p1 = 0.008). The mean TBL_pre_ and TBL_pod1_ (375.5 ± 242.0 ml; 318.0 ± 183.4 ml) were lower than TBL_post_ (488.2 ± 271.1 ml), p2 and p3 < 0.001. The mean HBL_pre_ (375.5 ± 242.0 ml) were higher than HBL_pod1_ (226.5 ± 163.2 ml), p1 < 0.001. The mean HBL_pod1_ was lower than HBL_post_ (226.5 ± 163.2 ml; 396.7 ± 254.0 ml), p3 < 0.001. Yet such differences were not detected between HBL_pre_ and HBL_post_ (375.5 ± 242.0 ml, 396.7 ± 254.0 ml, p2 = 0.361) (Table 3). HBL_pre_ was the major part of the peri-operative HBL.

**Table 3:** Comparison of pre- and post-operative blood loss.

**Variable**	**pre**	**pod1**	**post**	**p**	**p1**	**p2**	**p3**
**HBL(ml)**	375.5 ± 242.0	226.5 ± 163.2	396.7 ± 254.0	<0.001	<0.001	0.361	<0.001
**TBL(ml)**	375.5 ± 242.0	318.0 ± 183.4	488.2 ± 271.1	<0.001	0.008	<0.001	<0.001

p: p value of pre vs pod1 vs post; p1: p value of pre vs pod1; p2: p value of pre vs post; p3: p value of pod1 vs post; TBL: total blood loss; HBL: hidden blood loss.

## Discussion

As is mentioned before, peri-operative blood loss has been a threaten for elderly patients. However, previous researches were focused on blood loss during surgery and post-operative drainage which accounted for transfusion requirements but ignores significant HBL during the whole peri-operative period^10,16,17^.

Gradually some studies analyzed the HBL and hip fractures. Foss *et al*. assessed 546 hip fracture patients and hypothesized that hidden blood loss could originate from post-operative hemorrhage, anti-coagulation, and bleeding from other sources such as the gastrointestinal tract^10^. Cai *et al*. and Yu *et al*. demonstrated that large amount of post-operative hidden blood loss existed in elderly patients with intertrochanteric fractures treated by PFNA, nevertheless, they did not pay attention to the pre-operative hidden blood loss^11,12^. Yang *et al*. proved the peri-operative hidden blood loss of elderly patients with unstable intertrochanteric fracture treated by different intramedullary fixations, but the proportion of pre-operative HBL remains unknown^13^. In a retrospective study of 168 patients, Smith *et al*. conjectured that a significant proportion of the hidden blood loss related to hip fractures occurred prior to surgery^17^. Although Smith’s study changed an angle to analyze blood loss condition before surgery, the specific volume of HBL was not calculated.

On the other hand, the formula of total red blood cell loss, TRBCL (L) = PBV* (Hct_pre-operation_ − Hct_post-operation_), was one of the key procedures to calculate HBL. The Hct_pre-operation_ in previous studies were the data recorded on admission day or pre-operative day without a specific description. If the waiting time to operation was more than a day, using the formula HCT_post-operation_ minus Hct_pre-operation_ actually finally got the result of total HBL from admission to post-operation days which was regarded as the post-operative HBL. In this study, to calculate pre- and post-operative HBL accurately, blood routine of different time points was recorded, blood loss of different periods were calculated respectively as mentioned before.

Current evidence-based clinical research has revealed that PFNA can minimize the risk of implant-related complications caused by DHS and provide angular and rotational stability and better biomechanical stability than DHS which allows early mobilization and weight bearing of osteoporotic elderly patients^18–22^. Whereas the relatively simpler surgical procedure, shorter operative time and less intra-operative visible blood loss make it easy to ignore the HBL^23^. In addition, the causes and mechanism of hidden blood loss are not yet clear, some studies speculated the process of expanding the medullary cavity could lead to internal bleeding^11–13,16^. As the elderly patients were usually accompanied with expectable senile osteoporosis and the diameter of the medullary cavity were usually extended and more than 10 mm^24–26^, in this retrospective study, all the patients underwent a PFNA surgery without a reaming process.

It is obvious that the patients had a large drop in Hb before surgery and interference factors which could result in Hb decrease like hematopathy, laboratory results of bleeding disorders, taking drugs that possibly impact blood coagulation, gastrointestinal hemorrhage were also excluded. We hypothesized this drop could originate from the pre-operative HBL and attempt to quantify the HBL prior to the surgery.

This study revealed that the visible blood loss (VBL, 91.5 ± 54.6 ml) was much less than the actual peri-operative total blood loss (TBL_all1_, 693.5 ± 359.6 ml; TBL_all3_, 863.8 ± 429.9 ml), a large amount of hidden blood loss still existed that it was more than 80% of total blood loss and four times more than visible blood loss. But the major proportion of hidden blood loss occurred before surgery. The mean HBL_pre_ was 375.5 ± 242.0 ml, which was significantly higher than HBL_pod1_ (226.5 ± 163.2 ml). Although HBL_post_ (396.7 ± 254.0 ml) was higher than HBL_pre_, it was not statistically significant (p = 0.361). Thus, even patients underwent the surgery, the HBL of 3 days post-operation was still no more than the pre-operative HBL. Furthermore, fracture union does not often happen soon in a few days after surgery. So, it implies that the calculated post-operative hidden blood may contain the amount caused by the initial trauma. Transfusions based on the post-operative Hb level could not only generate considerable costs, but also enhance the risk of transfusion reactions, fluid overload, and wound complications for elderly patients^27^. Therefore, early identification and management for elderly patients with intertrochanteric fractures at risk of anaemia are essential.

There are several limitations in this study. Firstly, we didn’t compare the data of non-surgery and patients underwent reamed PFNA. On the one hand, it was difficult to obtain the complete data of patients with non-surgical treatment because clinical intertrochanteric fractures had been treated with surgery in recent years. On the other hand, the unreamed PFNA was a mature treatment in our hospital, as we found the Hb drop before surgery, we wanted to make our first step to study the HBL of different periods in these patients who underwent the unreamed PFNA surgery. Further studies with comparison about the patients of non-surgery, reamed and unreamed PFNA would be performed with a lager samples to research whether the reaming process carry a substantial HBL. Secondly, the results of the calculated values were not totally accurate. The precise calculation of blood loss depends on the accuracy of haematocrit at admission and after surgery, the accurate measurement of visible blood loss and estimated patient’s blood volume. A dehydration secondary to the initial trauma and the delayed redistribution of red blood cells due to insufficient fluid or acute blood loss can cause falsely high haematocrit values. Then the rehydration after admission may lead to an underestimation of the post-operative haematocrit values. So, the blood loss may be overstated according to the calculations. However, the formula had been applied in studies published in high-quality international journals which was confirmed reliable and practical. In further studies, we will perform a more precise recording and calculation of blood loss.

## Conclusions

The majority of peri-operative HBL of elderly patients with intertrochanteric fractures treated by unreamed PFNA occurred before surgery. The peri-operative HBL was mainly associated with the initial trauma rather than the operation. When managing elderly patients with intertrochanteric fractures, it is necessary to consider the pre-operative significant blood loss and identify elderly patients at risk of anaemia so as to provide appropriate pre-operative medical managements.

### Data availability statement

The datasets generated and analyzed during the current study are available from the corresponding author on reasonable request.
